# A comparative study of methods for determining Intravoxel incoherent motion parameters in cervix cancer

**DOI:** 10.1186/s40644-020-00377-0

**Published:** 2021-01-14

**Authors:** Xue Wang, Jiao Song, Shengfa Zhou, Yi Lu, Wenxiao Lin, Tong San Koh, Zujun Hou, Zhihan Yan

**Affiliations:** 1 Department of Radiology, The Second Affiliated Hospital and Yuying Children’s Hospital of Wenzhou Medical University, 109 Xueyuanxi Road, Wenzhou, 325027 China; 2 Department of Oncologic Imaging, National Cancer Center, Singapore 169610 and Duke-NUS Graduate Medical School, Singapore, 169547 Singapore; 3 Suzhou Institute of Biomedical Engineering and Technology, Chinese Academy of Sciences, Suzhou, 25163 China

**Keywords:** Cervical cancer, Intravoxel incoherent motion, Diffusion-weighted MRI

## Abstract

**Background:**

To compare different fitting methods for determining IVIM (Intravoxel Incoherent Motion) parameters and to determine whether the use of different IVIM fitting methods would affect differentiation of cervix cancer from normal cervix tissue.

**Methods:**

Diffusion-weighted echo-planar imaging of 30 subjects was performed on a 3.0 T scanner with *b*-values of 0, 30, 100, 200, 400, 1000 s/mm^2^. IVIM parameters were estimated using the segmented (two-step) fitting method and by simultaneous fitting of a bi-exponential function. Segmented fitting was performed using two different cut-off *b*-values (100 and 200 s/mm^2^) to study possible variations due to the choice of cut-off. Friedman’s test and Student’s t-test were respectively used to compare IVIM parameters derived from different methods, and between cancer and normal tissues.

**Results:**

No significant difference was found between IVIM parameters derived from the segmented method with *b*-value cutoff of 200 s/mm^2^ and the simultaneous fitting method (*P*>0.05). Tissue diffusivity (*D*) and perfusion fraction (*f*) were significantly lower in cervix cancer than normal tissue (*P*< 0.05).

**Conclusions:**

IVIM parameters derived using fitting methods with small cutoff *b*-values could be different, however, the segmented method with *b*-value cutoff of 200 s/mm^2^ are consistent with the simultaneous fitting method and both can be used to differentiate between cervix cancer and normal tissue.

## Background

Cervix cancer is the third most commonly diagnosed cancer and the fourth leading cause of cancer death in women worldwide [1]. Intravoxel incoherent motion (IVIM) MR imaging has been explored as a potential imaging biomarker for characterizing cervical cancer and monitoring of neoadjuvant chemotherapy in cervical cancer [2–4]. IVIM imaging has also been widely utilized in other organs, such as differentiation of tumor stages and histological types in head and neck cancers [5, 6], differential diagnosis of low and high-grade gliomas [7], as well as tumors in abdominal and pelvic organs, including liver [8–10], pancreas [11, 12], kidney [13–15], and prostate [16, 17].

The IVIM model differentiates water movement in tissues into diffusion in extravascular space and perfusion-related pseudo-diffusion in blood vessels, represented by the diffusion coefficient (*D*) and pseudo-diffusion coefficient (D*), respectively [18]. In contrast to a single exponential model which only accounts for diffusion in the tissue interstitial and cells (extravascular space), a major advantage of the IVIM model is the ability to also account for tumor blood flow (by the pseudo-diffusion coefficient D*). As the malignant tumor relies on its blood supply for growth and progression, additional information on tumor microcirculation derived from IVIM imaging could potentially be used for diagnostic and prognostic purposes in the management of cancer. A bi-exponential relationship is used to describe diffusion-weighted signal *S*_*b*_ acquired at different *b-*values:

1 $$ \frac{S_b}{S_0}=\left(1-f\right)\exp \left(- bD\right)+f\kern0.5em \exp \left[-b\left(D\ast +D\right)\right] $$

where *f* denotes the perfusion fraction. Several studies have adopted a segmented (two-step) approach proposed by Luciani et al. [8] for fitting of the IVIM model. The initial step involves estimation of *D* by a simplified mono-exponential fit to the portion of the data with higher *b-* values, by specifying a cut-off *b*-value. Subsequently, with the value of *D* fixed, *f* and D* can be estimated by non-linear regression fitting with all acquired *b-*values. Considering that D* is much larger than *D*, the segmented approach assumes that the influence of D* on signal decay for *b*-values larger than a certain cut-off would be negligible compared with *D*. Different cut-off values (ranging from about 100–200 s/mm^2^) have been implemented in several studies involving various parts of the body [3, 5, 7, 8, 19–23], possibly due to the difference in perfusion characteristics in the various organs and tissue types. However, it is not clear whether IVIM results reported for a particular cancer could be affected by the use of different cut-off values in different institutions. Understanding whether a different cut-off would have significant effects on IVIM parameters could help improve the robustness of IVIM imaging for multi-center studies. It is therefore of interest to explore whether the segmented approach is robust to the choice of cut-off *b*-value and for a particular region of the body.

There is currently a lack of studies (no publication was found in a recent PubMed search) on whether different IVIM computation methods would affect its utility in the differential diagnosis of cancer. In this study, IVIM parameters obtained using 2 different cut-off *b*-values for segmented fitting and by simultaneous fitting of the bi-exponential function, were compared with the aim of exploring whether these different analysis methods would affect the ability of IVIM parameters in differentiating cervical cancer from normal tissue.

## Methods

### Patients and MR imaging protocol

A waiver of consent for retrospective analysis of imaging data was granted by the local institutional review board because of the anonymous nature of this study. Thirty-five consecutive female patients (mean age, 48.4 years; age range, 41–67 years) with clinical symptoms of cervical cancer (e.g vaginal bleeding, abnormal discharge or pelvic pain) were presented to our department between March 2016 and September 2017. Our diagnostic protocol for patients suspected with cervix cancer included both cervical biopsy and MRI examination. Among the 35 consecutive patients, biopsies of 24 patients revealed the presence of cervix cancer cells, while cancer cells were not found in the biopsies of 11 subjects. For these 11 subjects with negative biopsies, their MRI examinations also did not reveal any suspicious mass or lesion, and therefore, their MRI data were considered as part of the normal tissue dataset. However, MRI data of one subject with negative biopsy and one patient with positive biopsy were excluded because of significant motion artifacts. Furthermore, 3 patients with positive biopsy were excluded because no obvious mass or lesion could be identified on MRI (i.e lesions could be too small) and a region-of-interest (ROI) for cancer cannot be identified on the MR images. Consequently, only MRI data of 20 patients with positive biopsy and 10 subjects with negative biopsy were processed in this study. Twenty ROIs for cervical cancer were obtained from the 20 patients with positive biopsy (i.e one ROI for each patient). Only 10 ROIs for normal-appearing tissue can be identified among the 20 patients with positive biopsy (because some tumors were too extensive and obscured the outline of normal-appearing tissue), while another 10 ROIs for normal tissue were obtained from the 10 subjects with negative biopsy (one ROI for each subject). Cervix cancer was clinically staged according to the International Federation of Gynecology and Obstetrics classifications. The 20 patients with positive biopsy were classified into stage Ib (*n*=8), IIa (*n*=6) and IIb (*n*=6). All patients were confirmed histopathologically and hysterectomy was performed for patients with cervix cancer.

MR imaging was performed on a 3.0 T scanner (Discovery™ MR750w, General Electric, USA) using an 8-channel torso phased-array coil. Routine clinical MRI sequences included a transverse fast spin-echo T1-weighted sequence (repetition time/echo time = 550~700/7~10 ms; field of view = 320 × 320 mm; matrix size = 512 × 512; slice thickness = 5.0 mm; intersection gap = 6 mm), and a short time inversion recovery (STIR) T2-weighted sequence (repetition time/echo time = 3000~4000/70~80 ms; field of view = 256~320 × 256~320 mm; matrix size = 512 × 512; slice thickness = 5.0 mm; intersection gap = 6~7 mm). Late contrast-enhanced T1-weighted (repetition time/echo time = 4/2 ms, flip angle = 13^°^, field of view = 280 × 280 mm, matrix size 512 × 512, slice thickness = 3 mm) images were acquired in the sagittal plane about 5 min after contrast administration.

Before administration of contrast agent, diffusion-weighted imaging (DWI) was performed using a single-shot spin-echo echo-planar imaging sequence in the axial plane with the following parameters: repetition time/echo time = 2400/74 ms, field of view = 360 × 360 mm, matrix size = 256 × 256 mm, number of slices = 10, slice thickness = 5.0 mm, intersection gap = 5 mm, number of averages=3. DWI data was acquired as trace-weighted images using 3 orthogonal-direction diffusion gradients, with parallel imaging (ASSET, acceleration factor = 2) to reduce possible imaging artifacts due to movement and eddy-currents. We acquired 6 *b*-values as follows: 0, 30, 100, 200, 400, 1000 s/mm^2^. These *b*-values were selected based on considerations for the prudent use of imaging time and the minimal requirements for estimation of IVIM parameters (i.e at least 2 *b*-values more than 200 s/mm^2^ for estimation of *D*, and at least 2 *b*-values less than 100 s/mm^2^ to adequately depict the initial rapid decrease). The total scan time for DWI was approximately 2.36 min. These aspects of the relative dominance of D and D* on opposite extremes of b-values is also related to the assumption of negligible water exchange between blood and tissue in the IVIM model (see Discussion on limitations of standard Gaussian and non-Gaussian IVIM).

### Image analysis

For each patient, ROIs for cervix cancer and normal-appearing cervix tissue were manually delineated on multiple slices of the DW images by a radiologist with 3–4 years of experience in gynecologic imaging. Routine T1-weighted, T2-weighted (STIR with matrix 512 × 512) and contrast enhanced scans were used for cross-referencing to confirm the location and size of lesions when the DW images were evaluated. Tumor size in these patients ranged from 1.6–4.9 cm, which can be adequately covered by the 10 imaging slices in the DW images. For cervix cancers, ROIs were positioned on the solid area of the lesions on *b* = 1000 s/mm^2^ DW images with the exclusion of cystic and necrotic regions. ROIs for the corresponding normal outer cervix were placed far from the cervix cancer and cervical cysts on *b* = 0 s/mm^2^ images.

Voxel-level fitting of the DW signal *S*_*b*_ with the IVIM model (Eq. (1)) was performed using Matlab™ (The Mathworks, Natick, MA). In the segmented fitting approach [19], *D* was first estimated by a simple linear fit of the data corresponding to *b-*values ≥ cut-off, as described by a single exponential.

2 $$ \frac{S_b}{S_0}=\exp \left(- bD\right) $$

Subsequently, with the value of *D* fixed, *S*_0_, *f* and D* were estimated by fitting Eq. (1) to data acquired at all *b*-values using nonlinear regression (‘lsqcurvefit’, Matlab™In this study on cervix cancer, two values of cut-off were implemented, i.e. *b-*value ≥ 100 s/mm^2^ and *b-*value ≥ 200 s/mm^2^, which are denoted as method 1 (M1) and 2 (M2), respectively. The two values were selected because cut-off values used in IVIM studies usually ranged from about 100–200 s/mm^2^ [21–23]. The method of simultaneous fitting is denoted as method 3 (M3), and all parameters (*S*_0_, *D*, *f* and D*) were concurrently adjusted in the fitting of Eq. (1) by nonlinear regression. It is noted that the DW image acquired for *b*=0 can be used as an estimate of *S*_0_, thus reducing the number of fitting parameters to 3 (*D*, *f* and D*). However, any noise in the *b*=0 DW image would then be propagated into the parameter estimates for *D*, *f* and D*. Alternatively, if more than 4 *b-*values have been acquired (i.e with the number of acquired *b-*values more than or equal to the number of estimated parameters = 4), *S*_0_ can be estimated as one of the fitting parameters, and the estimates for all 4 parameters may improve with the increase in number of *b-*values acquired. In this study, the DWI data consists of 6 *b-*values and all 4 parameters (*S*_0_, *D*, *f* and D*) are simultaneously adjusted in M3. Similarly, in the second step of M1 and M2, parameters *S*_0_, *f* and D* were adjusted using data from all 6 *b-*values, but with the value of *D* constrained to its estimated value in the first step.

### Statistical analysis

In each patient study case, more than one ROI could be identified for cancer or normal tissue on multiple slices. The median parameter value of all voxels within the tumor ROIs of each patient is taken as a representative statistic of tumor in the patient. The median values of the fitted parameters (*D*, *f* and D*) were used because the median is more robust to outliers (that could occur during data-fitting) than the mean. Similarly, the median parameter value within all normal tissue ROIs of each patient is taken as the representative statistic for normal tissue in the patient. Friedman’s test was used to test whether the choice of fitting method had a statistically significant impact on the estimated parameter values in both cancer and normal tissues. The Bland-Altman plot was utilized to analyze the agreement between any two fitting methods. For each fitting method, the Student’s t-test was used to compare median parameter values in cervical cancer and normal tissues. All statistical analysis was performed using SPSS software 18.0 (Chicago, IL, USA) and *P* < 0.05 was considered statistically significant.

## Results

**Fig. 1:**
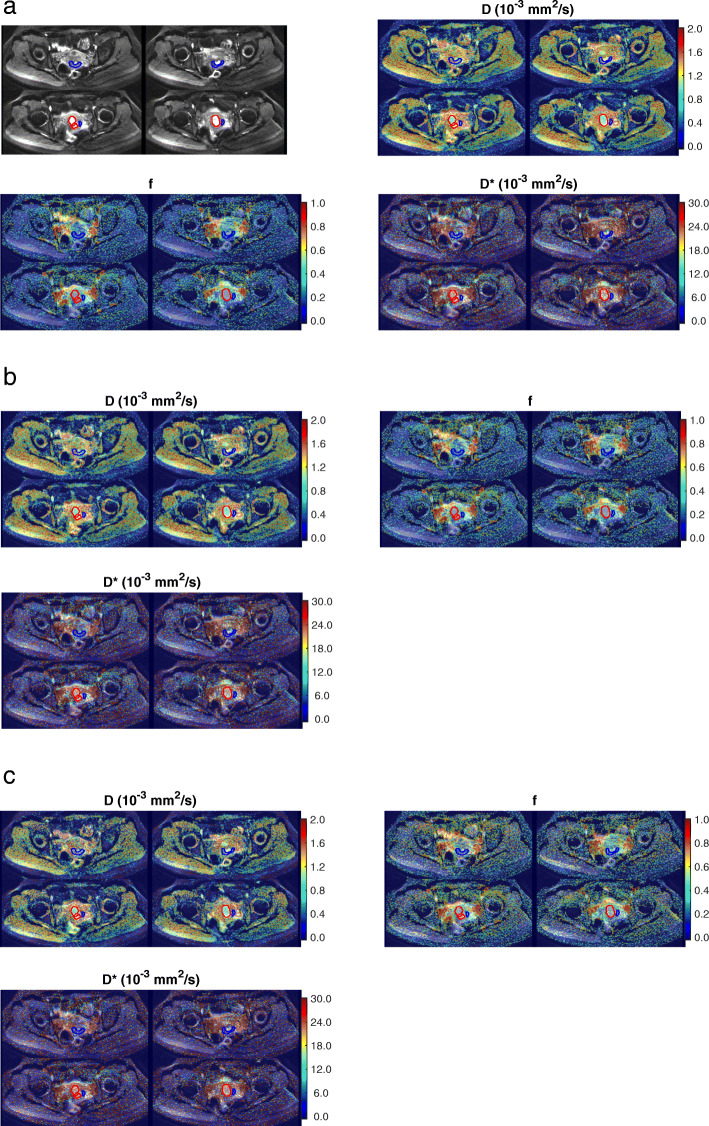
Example of a patient case with cervix cancer. Regions-of interest (ROIs) for cancer and normal appearing tissues are outlined in red and blue, respectively. **a** ROIs on the mean DW image and IVIM parameter maps generated from M1. **b** ROIs on the IVIM parameter maps generated from M2. **c** ROIs on the IVIM parameter maps generated from M3

**Fig. 2:**
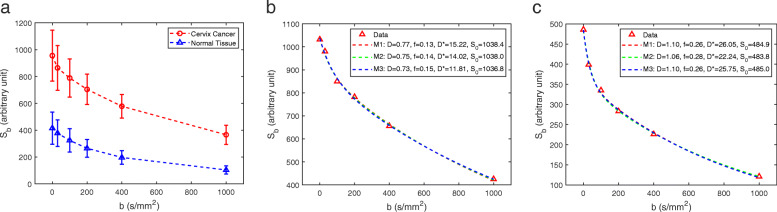
**a** Mean diffusion-weighted signal *S*_b_ (error bars denote standard deviation) in the cancer and normal tissue ROIs for the same patient shown in Fig. 1. **b** Fitting of a cancer voxel by all three methods. In the legend, units for *D*, *f*, D*, *S*_0_ and sum of squared error (SSE) are, respectively, mm^2^/s, unitless, mm^2^/s, arbitrary unit (a.u.) and (a.u.)^2^. **c** Fitting of a normal tissue voxel by all three methods

Example of a patient with cervical cancer is shown in Fig. 1 together with the IVIM parameter maps generated using the three fitting methods. The average DW signal *S*_*b*_ within cancer and normal tissue ROIs of this patient are shown in Fig. 2(a). The DW signal curves in cervix cancer are generally higher than those in the normal cervix tissue for all *b*-values up to 1000 s/mm^2^. Examples of fitting a cancer and normal tissue voxel by all three methods are shown in Fig. 2(b) and (c), respectively. Both cervix cancer and normal tissue *S*_*b*_ curves can be well-fitted by a bi-exponential function, with steeper initial slopes corresponding to the lower *b*-values. Parameter values derived using the three methods are shown in Table 1.

**Table 1:** Median values of IVIM parameters in cervical cancer and normal tissue ROIs of each subject obtained using the three fitting methods (M1, M2 and M3)

	D (10^− 3^ mm^2^/s)						D* (10^− 3^ mm^2^/s)						f					
	Cancer			Normal Tissue			Cancer			Normal Tissue			Cancer			Normal Tissue		
Subject id	M1	M2	M3	M1	M2	M3	M1	M2	M3	M1	M2	M3	M1	M2	M3	M1	M2	M3
1	0.77	0.70	0.74	1.20	1.04	1.13	11.14	14.80	13.85	19.34	5.59	8.81	0.13	0.18	0.14	0.20	0.32	0.24
2	0.93	0.88	0.91	1.28	1.24	1.21	22.61	18.72	16.20	54.08	54.77	28.93	0.11	0.16	0.13	0.22	0.26	0.25
3	0.69	0.63	0.66	1.36	1.41	1.30	20.27	15.15	13.98	38.94	44.23	34.42	0.11	0.16	0.13	0.32	0.33	0.34
4	1.08	0.76	0.99	0.90	0.70	0.83	16.25	10.49	12.07	12.22	7.06	9.66	0.23	0.38	0.27	0.26	0.36	0.30
5	1.11	1.20	1.07	1.03	1.19	1.00	21.08	67.62	15.93	42.73	44.50	40.09	0.16	0.12	0.18	0.28	0.23	0.28
6	0.77	0.70	0.74	1.02	0.81	1.02	17.80	11.51	13.37	8.96	3.29	7.17	0.11	0.17	0.13	0.09	0.18	0.11
7	0.81	0.73	0.81	1.29	1.27	1.21	19.57	16.20	17.36	29.16	30.69	23.88	0.15	0.22	0.17	0.31	0.36	0.34
8	0.84	0.68	0.80	1.29	1.21	1.23	12.39	6.56	9.38	19.99	14.81	14.22	0.13	0.23	0.15	0.22	0.30	0.26
9	0.74	0.73	0.73	1.36	1.34	1.33	24.00	20.16	20.12	34.3	31.44	35.40	0.10	0.11	0.11	0.22	0.23	0.24
10	0.83	0.81	0.81	1.55	1.50	1.60	37.88	29.29	37.86	45.99	41.66	51.79	0.11	0.12	0.14	0.29	0.30	0.27
11	0.70	0.68	0.68				35.41	28.09	34.00				0.10	0.11	0.13			
12	0.65	0.63	0.64				47.34	40.42	45.46				0.11	0.12	0.13			
13	0.86	0.85	0.84				30.11	23.1	30.81				0.13	0.14	0.15			
14	0.92	0.90	0.89				28.92	21.09	29.80				0.13	0.14	0.16			
15	0.74	0.73	0.70				24.00	20.12	19.66				0.10	0.14	0.16			
16	0.81	0.85	0.67				11.73	14.6	6.92				0.18	0.16	0.23			
17	0.83	0.81	0.81				37.88	29.29	37.86				0.11	0.12	0.14			
18	0.80	0.77	0.75				23.56	18.04	18.45				0.12	0.13	0.19			
19	0.85	0.83	0.76				28.92	20.58	25.46				0.10	0.12	0.16			
20	0.86	0.84	0.79				18.04	12.69	10.85				0.09	0.10	0.15			
21				1.63	1.57	1.64				31.33	28.46	36.58				0.32	0.34	0.37
22				1.46	1.46	1.37				19.96	16.96	13.50				0.23	0.14	0.14
23				1.49	1.42	1.32				16.12	17.31	16.14				0.25	0.19	0.25
24				1.49	1.44	1.22				16.13	12.62	12.13				0.13	0.16	0.22
25				1.06	1.09	0.98				19.28	20.49	17.49				0.21	0.21	0.30
26				1.53	1.46	1.41				16.64	12.59	13.67				0.25	0.28	0.32
27				1.55	1.50	1.60				45.99	41.66	51.79				0.29	0.30	0.27
28				1.28	1.23	1.19				21.16	15.47	18.49				0.24	0.26	0.38
29				1.36	1.30	1.39				11.51	8.80	20.81				0.15	0.17	0.32
30				1.27	1.20	1.07				17.52	12.70	12.97				0.22	0.26	0.39
Overall Mean±SD	0.83 ±0.12	0.79 ±0.13	0.79 ±0.11	1.32 ±0.20	1.27 ±0.23	1.25 ±0.22	24.45 ±9.65	21.93 ±13.29	21.47 ±10.93	26.07 ±13.32	23.26 ±15.30	23.40 ±13.87	0.13 ±0.03	0.16 ±0.06	0.16 ±0.04	0.24 ±0.06	0.26 ±0.07	0.28 ±0.07

### Comparison of IVIM parameters derived using different fitting methods

Table 2 summarized the results of Friedman’s test for comparing IVIM parameters obtained using different fitting methods, for both cancer and normal tissue ROIs. Firstly, it was noted that results for comparison of all three methods, pairwise comparison of M1 and M2, as well as pairwise comparison of M1 and M3, showed significant difference (with *P*< 0.05), except of the parameter D*. Secondly, it can be observed from Table 2 that no significant difference was detected in the parameter values estimated using M2 and M3 (*P*> 0.5).

**Table 2:** Results of Friedman’s test for comparison of IVIM parameters derived from all three fitting methods (M1, M2, M3) and for pairwise comparison of the fitting methods. A *P* value < 0.05 indicates significant difference in parameters obtained using the methods compared, which was highlighted by *

Statistical Test	Models Compared	D		D*		f	
		Cancer	Normal Tissue	Cancer	Normal Tissue	Cancer	Normal Tissue
Friedman’s test	M1, M2, M3	*P=0.032	*P=0.007	*P=*0.341	*P=*0.86	*P=0.005	*P=0.009
	M1 vs M2	*P< 0.001	*P=0.027	*P=0.001	*–*	*P=0.001	*P=0.017
	M1 vs M3	*P< 0.001	*P=0.006	*P=0.008	*–*	*P< 0.001	*P=0.005
	M2 vs M3	*P=*1.000	*P=*1.000	*P=*1.000	*–*	*P=*0.805	*P=*1.000

**Fig. 3:**
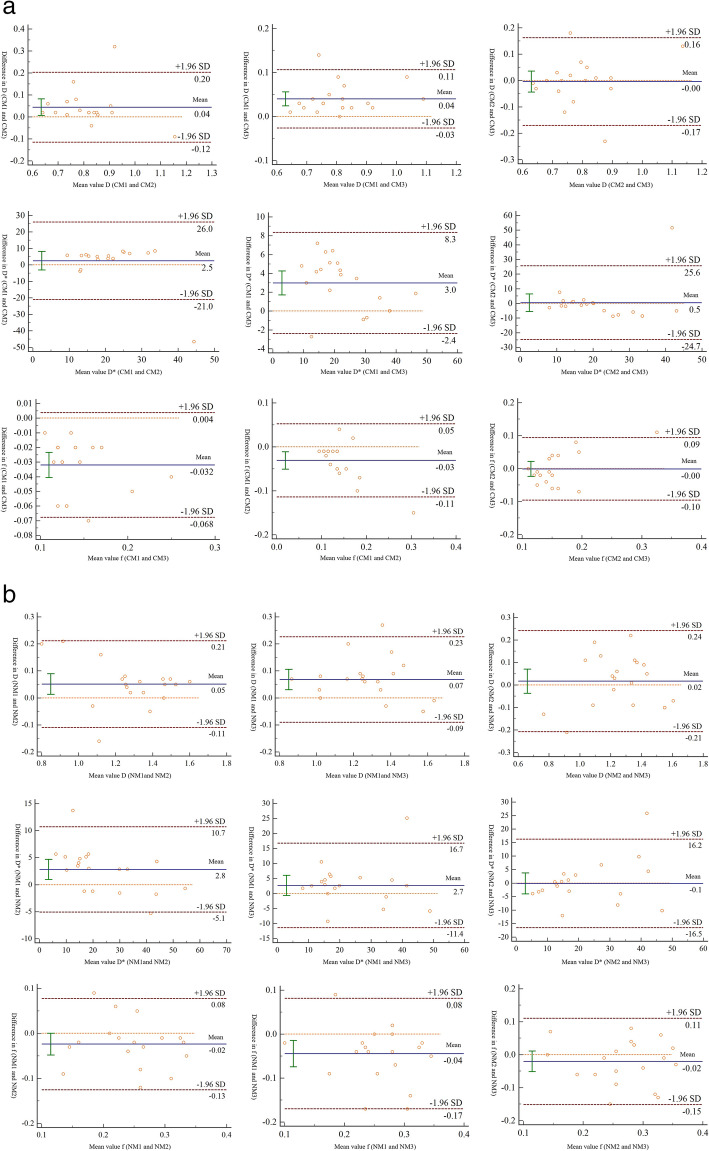
**a** Bland-Altman plots for D, D* and f derived from the three fitting methods in cervix cancer (CM1, CM2 and CM3). **b** Bland-Altman plots for D, D* and f derived from the three fitting methods in normal cervix tissue (NM1, NM2 and NM3)

Bland-Altman plots for pairwise comparison of IVIM parameters derived from the three fitting methods are shown in Fig. 3 for cervix cancer (a) and normal cervix tissue (b). While the mean differences of parameters were largely close to zero, there were evident disparities in some of the Bland-Altman plots, such as D* estimated by M1 and M3 in cancer, D* estimated by M1 and M2 in normal tissue, f estimated by M1 and M3 in both cancer and normal tissue, f estimated by M1 and M2 in cancer, as well as D estimated by M1 and M3 in both cancer and normal tissue. Evidently, only M2 and M3 showed no significant difference in all IVIM parameters for both cancer and normal tissue (Table 3).

**Table 3:** Results of Bland-Altman test for evaluating the significance of mean differences in IVIM parameters derived from pairwise comparison of the fitting methods (M1, M2, M3). * indicates significant difference (at significance level 0.05) between methods being compared

Statistical Test	Models Compared	D		D*		f	
		Cancer	Normal Tissue	Cancer	Normal Tissue	Cancer	Normal Tissue
Bland-Altman	M1 vs M2	*P=0.026	*P=0.012	*P=*0.360	*P=0.006	*P=0.004	*P=*0.052
	M1 vs M3	*P<0.0001	*P=0.001	*P=0.0001	*P=*0.113	*P<0.0001	*P=0.006
	M2 vs M3	*P=*0.835	*P=*0.527	*P=*0.875	*P=*0.940	*P=*0.927	*P=*0.186

*IVIM* intravoxel incoherent motion, *M1* method 1, *M2* method 2, *M3* method 3

### Comparison of IVIM parameters between cervix cancer and normal tissue

Results of Student’s t-test comparing IVIM parameters between cancer and normal tissue (Table 4) showed that *D* and *f* values were significantly different between cervix cancer and normal cervix tissue (*P* < 0.05), for all fitting methods. D* was not significantly different between cervix cancer and normal cervix tissue, for all fitting methods (*P* > 0.5).

**Table 4:** Results of Student’s t-test for comparing IVIM parameters (from Table 1) between cervix cancer and normal tissue for each fitting method (M1, M2, M3). A *P* value < 0.05 indicates significant difference in the parameter between cancer and normal tissue, which was indicated by *

	M1	M2	M3
D	*P=0.000	*P=0.000	*P=0.000
D*	*P*=0.662	*P*=0.771	*P*=0.628
f	*P=0.000	*P=0.000	*P=0.000

## Discussion

For *b*-values ≤ 1000 s/mm^2^, diffusion-weighted signal *S*_*b*_ curves (as a function of *b*-values) within cervix cancer are generally higher than those in normal cervix tissue, and both *S*_*b*_ curves can be well-described by a bi-exponential function. IVIM theory postulates that the two exponential functions can be attributed to water diffusion in the extravascular space and perfusion-related pseudo-diffusion in blood vessels, which are characterized by *D* and *D* * respectively, weighted by the perfusion fraction f. The present study suggested that with the proper choice of the cutoff *b*-value (i.e 200 s/mm^2^), consistent estimates of these IVIM parameters could be obtained by the method of segmented fitting and the method of simultaneous fitting, for both cervix cancer and normal tissues.

The segmented approach separately estimates the IVIM parameters in two steps, each involving a different signal model, given separately by Eqns. (1) and (2). The first step involves certain assumptions: D* is typically about an order larger than *D*, and the effect of D* on the DWI signal *S*_b_ would be weaker compared to *D* at higher *b*-values. Assuming that *S*_b_ would be dominated by *D* after a certain cutoff *b*-value, the first step only estimates *D* using *b-*values ≥ cut-off and a linear fit to the mono-exponential model (Eq. 2). The second step estimates the remaining 3 parameters (*S*_0_, *f* and D*) by a bi-exponential model (Eq. 1) using all acquired *b-*values while keeping *D* constrained at its estimated value obtained in the first step. Therefore, one may view segmented fitting as a form of constrained-fitting method with one of the parameters (*D*) constrained at a pre-determined value (obtained using a mono-exponential fit). Theoretically, although the method (M3) which allows for simultaneous fitting of all parameters (i.e all parameters are freely adjusted) could require more computation time than constrained fitting, simultaneous fitting should yield better goodness-of-fit compared with constrained-fitting and can be used as a reference for comparing with the other methods (M1 and M2). A measure of the goodness-of-fit can be given by the sum of squared error (SSE) between data and model fitting, with a smaller SSE denoting a better fit. Figures 2(b) and (c) show that M3 consistently yielded smaller SSE than M1 and M2 for both cancer and normal tissue. However, under noisy conditions, apart from potentially faster computations, segmented-fitting may have the added advantage of yielding more stable results with the pre-determination and constrain of *D* during fitting [8].

Results of comparison between M1 and M2 (Tables 2 and 3) showed that the choice of cut-off values between 100 and 200 s/mm^2^ had significant impact on segmented fitting of cervix cancer and normal tissue S_b_ curves. A plausible reason is that the choice of cut-off could affect the estimation of *D* in the first step of segmented fitting (which involves only b-values ≥ cut-off), resulting in an error/bias in *D*. The subsequent fitting of the entire S_b_ curve in step 2 by adjusting the other IVIM parameters with a biased *D* kept constant, would then result in deviations in the other parameters. The findings of previous studies [9–11] showed that effects of perfusion on the measured apparent diffusion coefficient (ADC) were diminished at *b*-values higher than 100 s/mm^2^. The present study showed that it would be more appropriate to choose the cutoff at 200 s/mm^2^, in order to yield results consistent with the simultaneous fitting of all parameters.

The implication of this observation for IVIM imaging of cervix cancer is that acquisition of 2 or 3 *b* values ≥ 200 s/mm^2^ might be sufficient to estimate *D*, and it might be more worthwhile to acquire more *b*-values that are less than 200 s/mm^2^ to improve estimation of *f* and D*. Furthermore, if one wished to be more prudent with imaging time and acquisition of *b*-values, a minimal set of *b*-values for IVIM imaging of the cervix may consist of a combination of the following choices of 4 to 5 *b*-values: [*b* = 0, 20–100, 200, 800–1000 s/mm^2^]. However, a larger population study would be required to validate this suggestion.

Fitting methods M2 and M3 consistently yielded similar IVIM parameters that can be used to differentiate cervix cancer from normal tissue: *D* and *f* were significantly lower in cervix cancer than normal tissue (while D* showed no significant difference(*P* > 0.5)). These results not only suggest potential clinical usefulness of IVIM imaging for cervix cancer, but also indicate possible robustness of IVIM imaging for multi-center studies of cervical cancer as institutions employing different fitting methods might be able to draw similar conclusions.

A previous study comparing segmented fitting (with cut-off > 200 s/mm^2^) and simultaneous fitting of IVIM model in brain tissues have suggested significant differences in parameters obtained using these methods [24]. Several reasons that could complicate IVIM fitting in brain tissues were highlighted and discussed [24], including the increased number of outliers due to simultaneous fitting, near mono-exponential behavior of signal decay in certain brain tissues, and the presence of cerebrospinal fluid resulting in non-physiological fitting and partial volume effects [24]. In this study, tumor and normal tissue ROIs were carefully delineated to exclude cysts and necrotic regions, and hence IVIM analysis of these cervix tissues may be less complicated. Outliers (due to fitting) would inevitably exist for both segmented and simultaneous fitting because of noise in the data. However, they did not occur in exceedingly large numbers (i.e more than half the population of fitted voxels) and the median value should still be representative of the ROI.

Consistent with previous IVIM studies of cervical cancer [3, 25–29], our results showed that *D* and *f* were lower in cervical cancer than normal tissue. Lower values of *D* in tumors have been commonly attributed to increased cellularity and further hindrance of diffusional movement of water molecules in the extravascular space. The relatively lower values of perfusion fraction *f* in cervical cancer as compared with normal cervix tissue is seemingly concordant with the clinical observation that cervix cancer exhibited lower enhancement in contrast-enhanced scans. D* was not significantly different between cervix cancer and normal tissue (*P* > 0.5). This is possibly due to higher uncertainty in the estimation of D* [30–32]. Several studies in various regions of the body have shown that D* is associated with higher error and poor reproducibility due to its sensitivity to noise. Parametric maps of D* generated using all three fitting methods were generally more noisy than the corresponding maps of *D* and *f*, as shown in Fig. 1. Further studies in larger sample size are needed to investigate the value of D* for differentiating cervical cancer from normal tissue.

This study has the following limitations. A major limitation of the present study is the lack of a systematic quality assurance (QA) protocol for checking accuracy of DWI scans in our clinical scanner. A QA protocol for DWI serves to ensure and maintain the reliability of quantitative diffusion measurements [33, 34]. A recent QA study [33] among 44 MRI scanners in various institutions has reported a wide variability in ADC accuracy and spatial uniformity, depending on sequence implementation (i.e. gradient diffusion direction), spatial position and MRI scanner model. To ensure high level of accuracy and repeatability of results across institutions, Fedeli et al. [33] have proposed a specific QA protocol which should be adopted for single- and multisite studies as well as routine clinical practice. Such a QA protocol will be pursued in our institution for further DWI studies.

This study only implemented the standard IVIM model on cervical cancer data. As explained by Le Bihan [35, 36], recognizing that the ADC represents an approximation (assumed Gaussian behavior) of the complex diffusion process in biologic tissue by using a simple free diffusion equation, the standard (original) IVIM model attempts to extend the concept of ADC in tissues to include a component which accounts for blood flow in random vessels, i.e. a pseudo-diffusion coefficient (D*). An important assumption is that water exchange between blood and tissue is negligible during the encoding time (a hypothesis which still needs to be investigated), the two random processes are assumed decoupled and the resulting signal attenuation is simply the sum of the tissue and blood components (i.e bi-exponential) [35, 36]. In the absence of blood flow, the standard IVIM model would be reduced to the conventional mono-exponential (ADC) diffusion model [30, 35, 36]. Because D* is an order of magnitude larger than ADC in tissue and given the small fraction of blood (a few percent) compared to the overall tissue water content, the blood-flow driven IVIM signature typically appears as an initial rapid decay of the overall signal decay curve at small *b*-values (< ~ 100 s/mm^2^). In contrast, to better describe multi-*b*-value data above 1000 s/mm^2^, recent studies have further extended the standard IVIM model to account for non-Gaussian features which are readily observable at high *b*-values, i.e. IVIM with kurtosis or Non-Gaussian (NG-) IVIM [37, 38]. Effects of blood flow and diffusional kurtosis are more readily assessed on opposite extremes of the DWI dataset, at very low and high b-values, respectively. While NG-IVIM has shown feasibility in quantifying tumor microstructure [37, 38], the use of trace-weighted images can introduce substantial errors in the estimation of directional diffusional kurtosis (*K*) and diffusivity (*D*) values, i.e. the error in *K* varies non-monotonically with *K* and with the degree of diffusion anisotropy, and there is a trend of increasing absolute error with both increasing *K* and increasing degree of diffusion anisotropy [39, 40]. In this study, our interest was on the possible estimation of tumor blood flow (instead of kurtosis) and we have only applied the standard IVIM model on trace-weighted images with *b*-values ≤ 1000 s/mm^2^. Although the use of trace-weighted images might allow for an unbiased estimation of ADC under the assumption of a Gaussian diffusion model, the validity of approximating blood flow by a Gaussian-based pseudo-diffusion coefficient D* decoupled from tissue diffusion (i.e bi-exponential signal decay) in the standard IVIM model has not been clearly established [35, 36]. Further studies should be performed to explore these assumptions of the standard IVIM model, i.e. whether D* can be clearly correlated with blood flow measured by other modalities and to what extent this correlation might be affected by the use of trace-weighted images.

Another limitation of this study is the lack of correction for non-linearity of diffusion gradients, which can result in a systematic and spatially-dependent bias of the diffusion-encoding b-value [41, 42]. These errors in diffusion-encoding will result in spatially dependent inaccuracy of diffusion measurements, the extent of which varies between MRI systems and vendors [41, 42], resulting in lower concordance between measurements in multisite studies. Both phantom and human studies have shown that correction for gradient non-linearity is needed to derive accurate and reproducible diffusion measurements [41, 42]. In this study, we were unable to perform such corrections due to the lack of local expertise and relevant vendor support for our clinical scanner. Therefore, we should be cautious about the generalization of the present results to other scanners, and further validation of these results with the appropriate gradient non-linearity correction is encouraged.

Possible image distortions due to eddy-current effects and subject movement were not corrected for in the current study. These image distortions can cause mis-registration between DW images with different *b*-values, which results in errors in the estimated DWI parameters. Although software packages for image registration are widely available, one should be cautious not to apply these methods blindly [43]. A careful account of the image distortions (i.e stretch/compression) due to eddy-currents should also consider the change in signal intensity due to the change in shape of the voxel, i.e. when reversing the stretch/compression of the voxel, one should also modulate the signal intensity according to volumetric change [43]. Quantitative assessment of the consequences of neglecting to perform this signal modulation step on parameters derived from diffusion MRI is a topic of on-going research [43]. Also, as explained in Jones and Cercignani [43], when correcting for subject motion one must keep in mind that unlike most other forms of medical imaging data, DW images contain orientational information, and simply applying a rotation/geometric transformation to the DW-images might not be appropriate. Therefore, a ‘prevention is better than cure’ approach is adopted in the present study to minimize eddy current effects and motion artefacts at the acquisition stage by having a prudent choice of small *b*-values not more than 1000 s/mm^2^ and with parallel imaging, keeping the total acquisition time short and tolerable (2.36 min).

The number of patient cases analysed in this study is small and only consisted of stage I and II cancers. Patient cases with stage III and IV cervical cancers are uncommon in our institution because most patients would usually seek medical attention when the early symptoms of bleeding manifest at stage I and II. Nevertheless, these preliminary results are encouraging and further study with a larger and more diverse cohort is warranted. The optimal number and combination of *b* values for IVIM imaging of cervix cancer remain unknown. It is possible that acquisition of more *b* values < 100 s/mm^2^ may improve the accuracy of D* and establish potential clinical usefulness of D*.

## Conclusions

In conclusion, diffusion-weighted signal in both cervix cancer and normal tissue can be well-described by a bi-exponential function. IVIM parameters obtained using segmented fitting with cut-off value of 200 s/mm^2^ and by simultaneous fitting were not significantly different. The derived parameters *D* and *f*, may be used to differentiate between cervix cancer and normal cervix tissue.

## Data Availability

Applicable if necessary.

## Abbreviations

IVIM: Intravoxel Incoherent Motion
D: Tissue diffusivity
D*: Pseudo-diffusion coefficient
f: Perfusion fraction
ROI: Region-of-interest
M: Method
SSE: Sum of squared error
ADC: Apparent diffusion coefficient
QA: Quality assurance
NG: Non-Gaussian
K: Kurtosis
